# The triad of nasopharyngeal carcinoma pathogenesis: host genetics, viral infection, and environmental exposures

**DOI:** 10.3389/fonc.2026.1800709

**Published:** 2026-04-15

**Authors:** Indrajit Nath, Vandana Raphael, Neizekhotuo Brian Shunyu, Ayesha Siddiqui, Suvamoy Chakraborty, Biswajit Dey, Om Saswat Sahoo, Ruby Dhar, Subhradip Karmakar

**Affiliations:** 1 Department of Biochemistry, All India Institute of Medical Sciences, Guwahati, Assam, India; 2 Department of Pathology, North Eastern Indira Gandhi Regional Institute of Medical Sciences (NEIGRIHMS), Shillong, Meghalaya, India; 3 Department of E.N.T, All India Institute of Medical Sciences, Guwahati, Assam, India; 4 Department of E.N.T, North Eastern Indira Gandhi Regional Institute of Medical Sciences (NEIGRIHMS), Shillong, Meghalaya, India; 5 Department of Biochemistry, All India Institute of Medical Sciences, New Delhi, India; 6 Department of Biochemistry, Kasturba Medical College, Manipal Academy of Higher Education, Manipal, India

**Keywords:** environmental factors, Epstein-Barr virus, genetic polymorphisms, high-risk human papillomavirus, HLA microsatellites, host genetics, nasopharyngeal carcinoma, smoked and fermented foods

## Abstract

Nasopharyngeal carcinoma (NPC) are geographically restricted malignancies, exhibiting significantly higher incidence in specific regions and among certain ethnic groups, indicating strong genetic and region-specific etiological influences. The pathogenesis of these malignancies illustrates a multifaceted interaction involving host genetic predisposition, viral infections, as well as various environmental and lifestyle factors. This mini review brings together existing evidence regarding the molecular genetic factors influencing both EBV and HR-HPV-associated NPC, focusing on gene polymorphisms, viral biomarkers, and risk modifiers that vary across populations. Recurrent genetic associations involved polymorphisms in GSTM1, CYP1A1, XRCC1, TNF, HLA microsatellites, and xenobiotic metabolism genes, particularly CYP2A6. Markers linked to EBV, including LMP1, EBNA1, and circulating plasma EBV DNA, exhibited a consistent association with NPC susceptibility. Similarly, HR-HPV markers including E6 and E7 oncoproteins, p16INK4a overexpression, and HPV DNA detection serve as critical biomarkers for HPV-driven HNCs, but categorically these associations haven’t yet been discovered in regard to NPC. These findings classify NPC as a genetically modified, virus-related cancer and highlight the necessity for population-specific genetic risk assessment, EBV**/**HPV-derived biomarkers, and targeted preventative efforts.

## 1 Introduction

Nasopharyngeal carcinoma (NPC) is a malignancy originating from the epithelial lining of the nasopharynx and demonstrates one of the most striking geographic distributions among human cancers, with incidence rates varying more than 100-fold between high-risk and low-risk populations worldwide (1, 2). While rare in most parts of the world (incidence <1 per 100,000), NPC shows exceptionally high prevalence in Southeast Asia, esp. southern China, and certain regions of North-East India, particularly the states of Nagaland, Manipur, and Mizoram (1, 3). Epidemiological data indicate that the age-adjusted incidence rates of NPC in these regions are among the highest in the country. For instance, the Kohima district of Nagaland reports an age adjusted incidence rate of 19.4 per 100,000, comparable to high-incidence areas in Southern China (4). This contrasts sharply with other parts of India, where NPC incidence remains low. NPC is classified into three histological subtypes by the World Health Organization (WHO): keratinizing squamous cell carcinoma (Type I), non-keratinizing carcinoma (Type II), and undifferentiated carcinoma (Type III) (5). In endemic areas like North-East India, WHO Type III predominates, accounting for over 95% of cases (3), whereas Type I is primarily found in the West and has been epidemiologically associated with the human papillomavirus (HPV), though a definitive causal role requires further mechanistic substantiation (6).

While EBV-associated NPC dominates in endemic regions of Asia, high-risk Human Papillomavirus (HR-HPV), particularly HPV-16 and HPV-18, has emerged as the primary etiological agent for an increasing proportion of oropharyngeal squamous cell carcinomas (OPSCC) in Western populations (7). The incidence of HPV-positive OPSCC has risen dramatically over the past three decades in North America and Europe (8). Unlike EBV-associated NPC which predominantly affects the nasopharyngeal epithelium, HR-HPV demonstrates tropism for the lymphoepithelial tissue of the oropharynx, including the tonsils and base of tongue (9). This divergence in anatomical site preference reflects fundamental differences in viral biology, host tissue susceptibility, and environmental cofactors between these two virus-associated head and neck malignancies.

The comparative pathogenesis of EBV-NPC and HR-HPV-NPC reveals both shared and divergent molecular mechanisms. Both viruses employ strategies to evade host immune surveillance and promote cellular transformation, yet they utilize distinct oncogenic proteins and pathways. EBV’s latent membrane protein 1 (LMP1) mimics constitutively active CD40 receptor signaling, activating NF-κB, MAPK, and JAK-STAT pathways to promote cell proliferation and inhibit apoptosis (10). In contrast, HR-HPV’s E6 and E7 oncoproteins directly target the p53 and Rb tumor suppressors respectively (11, 12), leading to loss of cell cycle checkpoints and genomic instability. These mechanistic differences have important implications for understanding disease progression, prognosis, and therapeutic vulnerabilities in these two virus-associated cancers.

The northeastern states of India present a unique epidemiological setting for NPC research. Despite sharing genetic ancestry with other Asian populations, the region’s indigenous tribes (including the Manipuri, Naga, and Mizo ethnic groups) have developed distinct cultural practices and dietary habits that may contribute to their elevated NPC risk (3, 13). The etiopathogenesis of NPC is multifactorial. Genetic predisposition plays a crucial role, with studies indicating associations between NPC and specific human leukocyte antigen (HLA) genotypes prevalent in populations of East Asian descent (14). Environmental exposures, such as the consumption of smoked meats and fish, common in Northeast Indian diets, have been implicated in NPC development due to the presence of carcinogenic nitrosamines (4).

In the context of Northeast India, studies have demonstrated a high prevalence of EBV in NPC cases. For example, a study analyzing NPC biopsies from this region found EBNA1 and LMP1 expression in 92.5% and 90% of cases, respectively, underscoring the virus’s significant role in NPC pathogenesis in this population (15). The interplay between genetic susceptibility, environmental exposures, lifestyle factors and EBV infection in this population remains incompletely understood, though emerging evidence suggests region-specific risk patterns that differ from those observed in other high-risk areas like southern China (3, 13).

This mini review aims to synthesize current knowledge on the molecular pathogenesis of NPC, including genetic susceptibility factors and EBV and HPV interactions, and evaluate the contribution of region-specific environmental and lifestyle exposures to NPC risk. Relevant studies were identified from PubMed, and Scopus using keywords related to NPC, EBV, HPV, Genetic Polymorphisms and Environmental Risk Factors in Indian and East-Asian Populations. This helps identify gaps in current understanding and propose directions for future research for early detection strategies tailored to this high-risk disease. **Table 1** Summary of included studies with author, year, study location, design, population and key findings.

**Table 1:** Summary of included studies with author, year, study location, design, population and key findings.

Author (year)	Region	Sample	Key findings
Laskar et al., 2022 (16)	Nagaland, Mizoram, Manipur	29 cases, 26 controls	STEAP3_rs138941861 & JAG1_rs2273059 ↑risk; PARP4_rs17080653 & TGFBR1_rs11568778 protective; variants act via p53 pathway.
Chatterjee K et al. (2022) (17)	NE India	399 records; 130 tested vs 130 controls	High EBV prevalence linked to lifestyle factors and poor survival.
Chatterjee K et al. (2021) (18)	NE India	100 cases, 70 controls	BAX -248 G>A & BCL2–938 C>A associated with poor survival.
Laskar S et al. (2020) (19)	Global incl. NE India	Review	TP53_rs1042522, MDM2_rs2279744, CDKN1A_rs1059234 increase NPC risk.
Keppen C et al. (2020) (20)	Nagaland	128 cases, 180 controls	XRCC1 Arg280His, XPC Val499Ala, ERCC1 Cys8092Ala with tobacco/firewood exposure ↑NPC risk.
Singh SA et al. (2019) (21)	Nagaland, Mizoram, Manipur	123 cases, 189 controls	GSTM1/GSTT1 null + CYP1A1 T3801C ↑risk (OR up to 5.7); smoked meat & fermented fish amplify risk.
Roy Chattopadhyay N et al. (2019) 7	Nagaland, Manipur	70 cases, 70 controls	HLA polymorphisms impair EBV immune response.
Paul P et al. (2018) (1)	NE India	Review	GWAS indicates shared mechanisms with global NPC; highlights need for personalized care.
Roy Chattopadhyay N et al. (2017) (22)	Global	Review	Undifferentiated subtype predominant in high-incidence areas like Nagaland.
Sahu SK et al. (2016) (23)	NE India	70 cases, 70 controls	p53 codon72 Arg>Pro polymorphism associated with risk.
Singh SA et al. (2016) (24)	Nagaland, Mizoram, Manipur	100 cases, 90 relatives, 120 controls	XRCC1 Gln/Gln & XRCC2 variants ↑risk; lifestyle factors strongly synergistic.
Borthakur P et al. (2016) (15)	Nagaland	40 NPC tissues, 20 controls	EBNA1 & LMP1 expressed in >90% of cases.
Saikia A, et al. (2016) (25)	NE India	51 cases	EBV detected in 59% cases; linked to Naga ethnicity, smoked food, smoking.
Singh SA et al. (2015) (26)	NE India	170 HNC cases, 230 controls	CYP1A1 CC genotype with smoking/alcohol/betel quid markedly ↑risk (OR 6.1).
Lourembam DS et al. (2015) (3)	Manipur	105 cases, 115 controls	High EBV load in advanced NPC; risk from smoked meat, poor ventilation, alcohol.
Lakhanpal M et al. (2015) (14)	NE India	120 cases, 100 controls	HL003 (121) & D6S2704 (218) ↑risk; firewood, mud housing, alcohol significant; EBV RNA detected in 92%.
Choudhury JH et al. (2015) (26)	NE India	180 cases, 240 controls	CYP1A1 TC/CC + GSTM1 null ↑HNSCC risk 3.5-fold; strong gene–environment interactions.
Lakhanpal M et al. (2015) (21)	NE India	120 cases, 100 controls	TNF-β AG ↑risk; HSP70 (+2437) CC protective; alcohol, mud housing, firewood major risks.
Singh SA et al. (2014) (55)	NE India	70 cases, 100 controls	EBV plus smoked meat, fermented fish, tobacco, herbal medicine ↑NPC risk.
Ghosh SK et al. (2014) (13)	Nagaland, Mizoram, Manipur	64 cases, 88 relatives, 100 controls	Smoked/fermented foods, tobacco, alcohol, indoor cooking ↑NPC risk; GST null & EBV infection additively increase risk.
Sharma TD et al. (2011) (27)	Manipur	200 cases	Type III undifferentiated carcinoma 75%; high exposure to smoked meat, fermented foods, tobacco, firewood smoke.
Kataki AC, et al. (2011) (4)	NE India	Review	NPC incidence highest in Nagaland (19.4/100,000); highlights ethnic/genetic susceptibility.
Simons MJ (2011) (28)	Global incl. NE India	Review	Historical populations of East Asian ancestry migration & HLA A2-B46 associations explain incidence disparities.
Chelleng PK et al. (2010) (29)	Nagaland	47 cases, 94 controls	Smoked meat (aOR 10.8) and herbal nasal medicine (OR 21.9) strongly associated with NPC.

## 2 Profiling of nasopharyngeal cancer

### 2.1 Epidemiological profile and study demographics

NPC in North-East India demonstrates marked regional variation in incidence, with Nagaland’s Kohima district reporting the highest age-adjusted rate of 19.4 per 100,000, followed by Manipur and Mizoram (3.5 per 100,000), while Assam records the lowest incidence at 0.6 per 100,000, highlighting strong geographical and ethnic influences (4, 29). A consistent male predominance is evident across studies, with male-to-female ratios ranging from 1.46:1 to 3.8:1, suggesting possible hormonal or occupational exposure-related factors (14, 27). Additionally, NPC primarily affects individuals under 50 years of age, with 35.9% to 70.3% of cases occurring in this demographic, pointing toward early-onset disease potentially driven by genetic predisposition and early-life environmental exposures (3, 20).

Undifferentiated carcinoma (WHO Type III) is the predominant histological subtype of NPC, accounting for 57.6% to 88% of cases, especially in high-incidence regions, and demonstrates a strong association with EBV seropositivity, mirroring patterns observed in endemic zones such as Southeast Asia (25, 27). This subtype is notably prevalent among tribal and rural populations, reinforcing the link between EBV-driven oncogenesis and environmental or genetic susceptibility in these communities (7, 15). In contrast, keratinizing squamous cell carcinoma (WHO Type I) is less frequently reported (e.g., 55.7% in one study), suggesting alternative etiological mechanisms and possibly distinct molecular pathways in specific population subsets (23). Recent systemic review, however have stated about ~17% diagnosed NPC cases had previous HPV infections, suggesting a possible connection (30). HPV positivity was also more common in the keratinizing histologic subtype of NPC than in non-keratinizing subtypes (39% vs 16%), reinforcing the notion that EBV-positive and HPV-positive NPCs arise through distinct tumorigenic pathways (6).

### 2.2 Molecular and genetic associations

#### 2.2.1 Key genetic polymorphisms

Genetic susceptibility to NPC involves a complex interplay of oncogenes, tumor suppressors, DNA repair mechanisms, and detoxification pathways. Variants in *TP53* (rs1042522), particularly the Pro allele, are linked to impaired apoptotic function and heightened NPC risk, while *MDM2* (rs2279744) facilitates p53 degradation, further driving tumor progression (19). The STEAP3 Arg290His mutation (rs138941861) compromises oxidoreductase activity, potentially contributing to carcinogenesis (16). DNA repair gene polymorphisms such as XRCC1 Arg399Gln, especially the Gln/Gln genotype, show a 2.76-fold increased risk in smokers (24), and ERCC1 Cys8092Ala emerges as a strong independent predictor of NPC in Nagaland (OR = 1.89) [12]. Detoxification enzyme deficiencies, notably GSTM1 and GSTT1 null genotypes, significantly elevate NPC susceptibility (2.15–6.42-fold), particularly among tobacco users (13, 31). Moreover, individuals with CYP1A1 T3801C TC/CC genotypes combined with GSTM1 null status exhibit a synergistically increased risk, up to 6.42-fold, underscoring the role of gene-environment interactions in NPC pathogenesis (31). Metabolic enzyme gene deletions, particularly the GSTM1 and GSTT1 null genotypes, were found to increase susceptibility to NPC. The combination of both null genotypes magnified this risk significantly, especially in the presence of environmental carcinogens (21, 31) Furthermore, CYP1A1 T3801C and BCL2–938 C>A variants were implicated in poor clinical outcomes and enhanced tobacco sensitivity (18, 31).

#### 2.2.2 Aberrant cellular signaling in NPC

**Figure 1:**
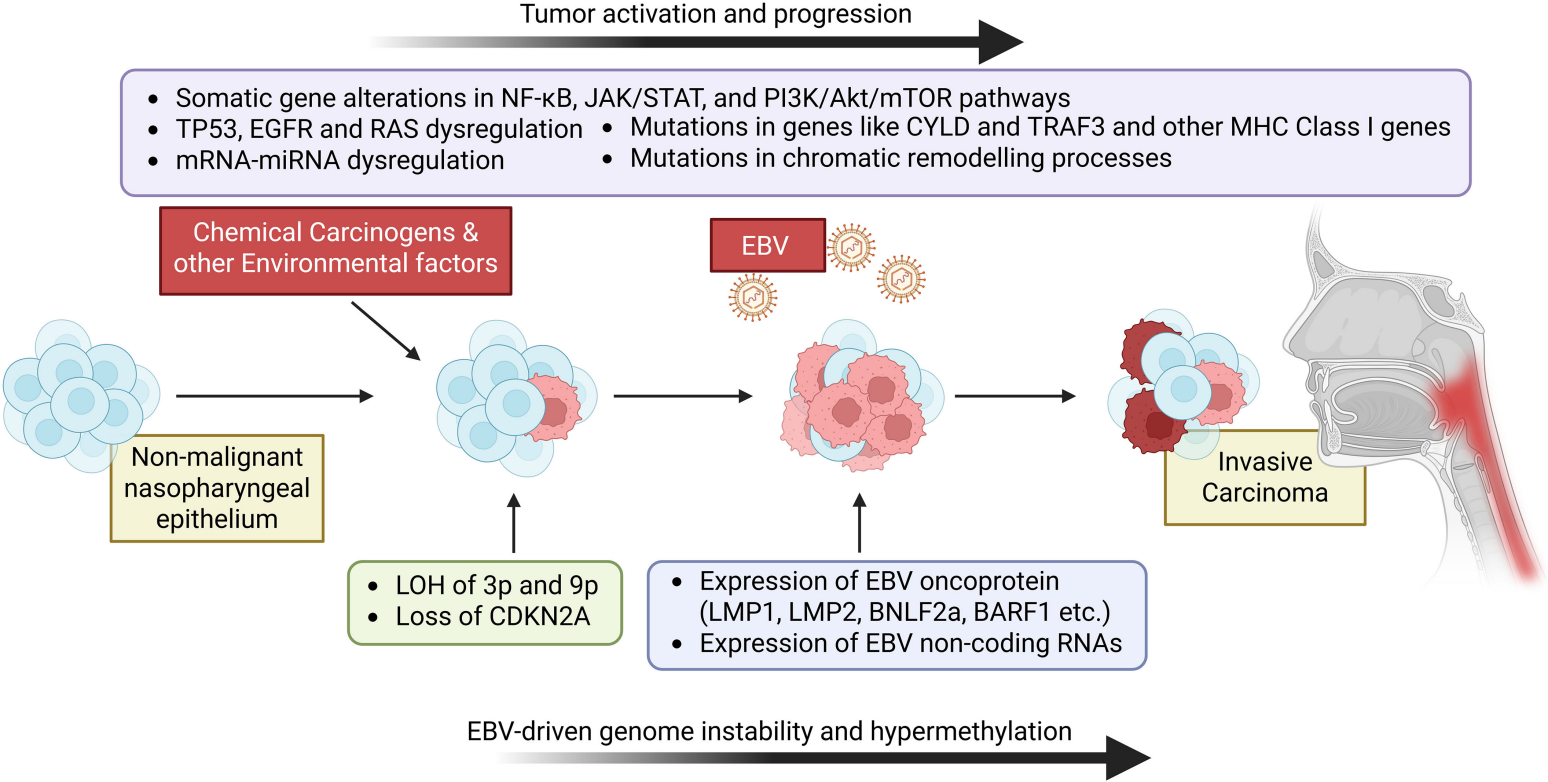
Molecular pathology of nasopharyngeal carcinoma The molecular pathology of nasopharyngeal carcinoma integrates EBV oncoproteins, dysregulated signaling, and RNA-network rewiring: latent LMP1/LMP2A chronically activate NF-κB, JAK/STAT, and PI3K/Akt/mTOR pathways; genetic lesions such as CDKN2A loss, PIK3CA amplification, and NF-κB regulator mutations amplify these cascades, while EGFR overexpression and Wnt/β-catenin activation drive growth and EMT. Concurrent mRNA-miRNA dysregulation (e.g., miR-205-5p upregulation targeting *TP53*, miR-34c-5p downregulation releasing *CCND1*) fine-tunes oncogenic programs, collectively promoting tumor proliferation, invasion, and immune evasion characteristic of NPC’s aggressive phenotype.

Aberrant cell signaling in NPC is driven by the concerted hijacking of multiple growth and survival pathways (**Figure 1**), most notably through EBV latent proteins and epigenetic dysregulation: EBV-encoded LMP1 chronically activates NF-κB, JAK/STAT, and PI3K/Akt/mTOR cascades to promote proliferation and inhibit apoptosis, while LMP2A sustains PI3K/Akt signaling and perturbs B-cell receptor mimicry in epithelial cells (32, 33). Concurrently, hypermethylation of Wnt antagonists such as DKK1 and SFRP unleashes β-catenin–dependent transcription, fueling epithelial–mesenchymal transition and stemness, and overexpression of EGFR/HER2 amplifies MAPK pathway output. Cross-talk with Notch and Hedgehog circuits further reinforces tumor-initiating cell phenotypes, and TGF-β–SMAD signaling cooperates with PI3K to drive invasion and immune evasion. Collectively, these aberrant signaling networks converge to sustain the malignant hallmarks of NPC, from rapid growth to metastatic spread (16, 19, 22).

Emerging evidence suggests HPV-EBV co-infection in NPC produces synergistic oncogenic effects. Co-infection creates additive or multiplicative signaling amplification. EBV LMP1 and HPV E6 both suppress *TP53* through distinct mechanisms, achieving near-complete p53 functional loss (12). Though for other cancers, HPV E7 and EBV EBNA3C both target pRb, with dual Rb inactivation drives more aggressive cell cycle dysregulation (11).

#### 2.2.3 Altered mRNA-miRNA network in NPC

In NPC, convergent mRNA–miRNA regulatory circuits orchestrate critical oncogenic processes. Deep sequencing and microarray profiling identified dysregulated miRNAs, viz. miR-34c-5p, miR-375, and miR-449c-5p are consistently downregulated, whereas miR-205-5p, miR-92a-3p, miR-193b-3p, and miR-27a-5p are upregulated, and their predicted targets include cell-cycle regulators (p53, CCND1, CDK6), pro-survival mediators (MET, BCL2), and extracellular matrix remodelers (MMP1/3, PLAU). Integrative analysis across five mRNA and two miRNA GEO datasets further constructed a kinase-TF-mRNA-miRNA network, uncovering 122 differentially expressed genes, 44 miRNAs, and hub nodes such as PTGS2, FN1, CD19, BMP2, and PIGR that coalesce signaling via NF-κB, PI3K/Akt/mTOR, and JAK/STAT pathways (34). Moreover, targeted exploration of NPC radio resistance networks revealed miRNA–mRNA pairs, most notably miR-203a-3p/BTK and miR-484/OLA1, offering mechanistic insights into therapy failure and potential biomarker axes for intervention (22, 34, 35).

#### 2.2.4 Whole genome sequencing to identify mutational burden in NPC

In NPC, whole‐exome sequencing (WES) has uncovered a characteristically low tumor mutational burden punctuated by recurrent alterations in tumor suppressors and oncogenic pathways, most notably CDKN2A deletions, mutations in NF-κB regulators (CYLD, TRAF3), and PIK3CA amplifications (1, 36), while familial NPC cohorts subjected to germline WES have revealed rare variants in DNA repair genes (BRCA2, NBN) and cell‐adhesion molecules (37). Integrating somatic mutation profiles with WGCNA modules has stratified NPC into molecular subtypes distinguished by unique mutational signatures and tumor microenvironment features, laying the groundwork for precision biomarker panels and targeted therapeutic strategies.

### 2.3 Multifactorial etiology

A multifactorial interplay of genetic, viral, and environmental determinants shapes NPC in North-East India. Immune response genes such as HLA microsatellites (D6S2704, HL003) with alleles like 218 and 121 have been linked to increased NPC susceptibility, likely due to compromised EBV immune surveillance (14). Protective genotypes, including TNF-β GG and HSP70 CC, contrast with the AG genotype, which elevates risk (38). EBV plays a central etiological role, with viral proteins EBNA1 and LMP1 detected in over 90% of NPC cases and high viral loads correlating with disease progression, from 1,200 copies/ml in early stages to 18,500 copies/ml in advanced stages [3,9]. The 30-bp deletion variant of LMP1, found in 89% of tumor biopsies, and EBER positivity (59%), especially among Naga individuals who consume smoked foods, suggest a region-specific viral mutation pattern (15, 25). Environmental exposures further compound risk: smoked meat (OR = 10.8), fermented fish (OR = 5.73) (21, 29), tobacco-betel quid chewing (OR = 7.0), and alcohol use (OR = 2.12) show strong associations, particularly when interacting with genetic variants like CYP1A1 TC/CC and GSTM1 null (31, 38) [19,22]. Additionally, household factors such as poor ventilation (OR = 3.79) and firewood use (OR = 12.07) significantly contribute to NPC pathogenesis, underscoring the need for integrated genomic and public health interventions (27, 38).

## 3 Multifactorial drivers of nasopharyngeal carcinogenesis

NPC development in Northeast India represents a paradigmatic example of multi-factorial carcinogenesis, involving intricate interactions between genetic susceptibility, viral infections, and environmental exposures. This complex pathogenetic model deviates from traditional single-factor causation theories, instead demonstrating how multiple risk factors synergistically contribute to malignant transformation through interconnected molecular pathways and cellular dysfunction mechanisms.

### 3.1 Genetic susceptibility framework with a population-specific genetic architecture

The Northeast Indian population exhibits a unique genetic landscape characterized predominantly by populations of East Asian ancestry, particularly evident in the Naga, Mizo, and other tribal communities. This genetic background shares significant similarities with Southeast Chinese populations, which demonstrate the highest global NPC incidence rates, suggesting common ancestral susceptibility loci that have been maintained through evolutionary bottlenecks and population migrations (4, 28). Specific HLA haplotypes prevalent in Northeast Indian populations may compromise immune surveillance mechanisms against viral infections and malignant transformation. The HLA-A*02:07 and HLA-B*46:01 alleles, have been associated with altered antigen presentation capabilities and reduced capacity for effective tumor immune recognition (39). Genetic analyses of mitochondrial DNA and Y-chromosome haplogroups reveal clustering with Southeast Asian populations, reinforcing evidence of ancient migration from the Yunnan and Guangxi regions of China into Northeast India via the Patkai and Arakan mountain ranges, a pattern echoed by Austroasiatic and Tibeto-Burman linguistic traces and Neolithic archaeological sites (40, 41). EBV molecular profiling further supports this connection, showing a predominance of EBV type A and unique LMP1 mutations with heightened oncogenic potential, closely resembling Southern Chinese strains (42, 43).

X-ray Repair Cross-Complementing (XRCC) Gene Polymorphisms: Critical mutations in XRCC1 and XRCC2 genes significantly compromise the cellular homologous recombination repair and base excision repair pathways (21, 24). These genetic variants reduce the cell’s capacity to correct DNA damage induced by environmental carcinogens, creating a permissive environment for malignant transformation.

1. XRCC1 Arg399Gln polymorphism: Reduces protein stability and DNA repair efficiency.
2. XRCC2 Arg188His variant: Compromises homologous recombination fidelity.
3. Cumulative effect: Multiple polymorphisms create synergistic repair deficiencies.

Together, these findings weave a comprehensive epidemiological narrative linking population-specific genetic susceptibility, DNA-repair polymorphisms, EBV strain variation, and historical migration to the elevated NPC burden observed in Northeast India.

### 3.2 Metabolic enzyme polymorphisms

Genetic variations in glutathione S-transferase enzymes, specifically homozygous deletions of GSTM1 and GSTT1 genes, result in the complete loss of essential phase II xenobiotic metabolism pathways. These enzymatic deficiencies impair the conjugation and elimination of electrophilic metabolites derived from environmental mutagens and carcinogens. Carriers of these deletion polymorphisms exhibit compromised detoxification of N-nitroso compounds, rendering them susceptible to increased genotoxic burden from dietary nitrosamines and environmental chemical exposures (21, 24).

Concurrent presence of chromosomal microsatellite instability and polymorphic variations in the genetic loci HL003 and D6S2704 reflects compromised genomic maintenance mechanisms. These molecular signatures indicate hereditary dysfunction in DNA mismatch repair surveillance systems, which synergistically interact with viral transformation pathways and chemical carcinogenesis to potentiate neoplastic progression (14).

### 3.3 EBV pathogenic mechanisms

EBV serves as the predominant infectious oncogenic driver in NPC pathogenesis, demonstrating near-universal presence in WHO Type III undifferentiated histological subtypes (**Table 2**) (15, 17, 25). The virus establishes persistent latent infection within nasopharyngeal epithelial cells through coordinated molecular hijacking mechanisms. Circulating cell-free EBV DNA concentrations and intratumoral viral genome copy numbers demonstrate positive correlation with disease stage progression, distant metastatic dissemination, and adverse clinical outcomes. Quantitative viral nucleic acid measurement provides both diagnostic utility and prognostic stratification for therapeutic monitoring applications.

**Table 2:** Epstein-Barr virus and molecular pathogenesis of NPC.

Marker/pathway	Findings	Population	Reference
EBNA1	Positive in 92.5% of NPC samples	Nagaland	Borthakur P et al. (2016) (15)
EBNA1	Positive in 40.8% of NPC samples	NE India	Chatterjee K et al.(2022) (17)
EBNA2	Positive in 0.8% of NPC samples	NE India	Chatterjee K et al.(2022) (17)
LMP1	Positive in 72.8% of NPC samples	Manipur, Nagaland, Mizoram	Singh SA et al. (2014) (55)
LMP1	Positive in 90% of NPC samples	Nagaland	Borthakur P et al. (2016) (15)
High EBV DNA Load	Associated with advanced NPC stage	NE India	Lourembam DS et al.(2015) (3)
PI3K, p53 Pathways	Variants observed in patients	NE India	Laskar S et al.(2020) (19)
EBER (CISH)	59% positivity; assoc. with smoked food and Naga ethnicity	NE India	Saikia A et al. (2016) (25)
EBV (*LMP1* gene)	EBV infection linked to GST null genotype & mtDNA alterations also 2.17 fold increased risk of NPC	Manipur,Nagaland,Mizoram	Ghosh SK et al. (2014) (13)
EBNA1	Positive in 87.86% of NPC samples	Manipur,Nagaland	Roy Chattopadhyay N et al. (2019) (7)
EBNA2	Positive in 9.94% of NPC samples	Manipur,Nagaland	Roy Chattopadhyay N et al.(2019) (7)

LMP1 functions as a constitutively activated pseudoreceptor mimicking tumor necrosis factor receptor signaling cascades. This viral oncoprotein drives sustained NF-κB transcriptional activation, promotes anti-apoptotic cellular reprogramming, and facilitates malignant transformation processes. LMP1 expression levels correlate with enhanced tumor aggressiveness and treatment resistance phenotypes (32).

EBV Nuclear Antigen 1 (EBNA1) maintains viral episomal genome persistence through replication licensing, induces chromosomal structural aberrations, and disrupts p53-dependent tumor suppressor checkpoints. EBNA1 detection confirms established viral latency programs and ongoing viral contribution to malignant phenotype maintenance. EBV infection establishes a pro-oncogenic microenvironment that facilitates secondary genetic alterations through sustained inflammatory signaling, reactive oxygen species generation, and immune recognition evasion (44, 45). The virus reprograms host cellular apoptotic thresholds, compromises DNA damage response fidelity, and disrupts cell cycle checkpoint integrity, thereby enabling progressive accumulation of transforming genetic lesions.

In addition, notable associations (as described above) of HPV have been made with respect to NPC. In contrast to HPV-driven oropharyngeal cancer, which is almost entirely associated with HPV16, HPV-positive NPC involves both HPV16 and HPV18 genotypes, though the clinical and biological significance of non-16 genotypes remains unclear (6).

### 3.4 Environmental carcinogenesis pathways and dietary carcinogen exposure of nitrosamine formation and metabolism

Traditional Northeast Indian dietary practices involve extensive consumption of preserved, smoked, and fermented foods that contain high concentrations of nitrosamines and polycyclic aromatic hydrocarbons (21, 24, 27). These compounds undergo metabolic activation to form DNA-reactive intermediates that cause specific mutational signatures in critical tumor suppressor genes. N-nitrosodimethylamine (NDMA) is Prevalent in smoked meats and fermented fish, N-nitrosodiethylamine (NDEA) is generated during food preservation processes (46, 47). Chronic alcohol consumption enhances nitrosamine formation through acetaldehyde generation and cytochrome P450 induction. Alcohol also compromises DNA repair mechanisms and promotes inflammatory responses that create a pro-carcinogenic tissue microenvironment (48).

Cigarette smoking and smokeless tobacco use introduce 4-(methylnitrosamino)-1-(3-pyridyl)-1-butanone (NNK) and N-nitrosonornicotine (NNN), which undergo metabolic activation to form DNA adducts in nasopharyngeal epithelial cells (3, 26). Betel Quid Components: Areca nut alkaloids (arecoline, arecaidine) undergo nitrosation reactions in the oral cavity to form N-nitrosoguvacoline and other carcinogenic nitrosamines. The alkaline environment created by lime promotes nitrosamine formation and enhances carcinogen absorption (49).

Last but not least, biomass Combustion Products and Chronic exposure to wood smoke from traditional cooking methods introduce formaldehyde, benzopyrene, and particulate matter into the respiratory tract (14). Poor ventilation in traditional housing amplifies exposure concentrations and duration. PM2.5 and ultrafine particles carry adsorbed carcinogens directly to nasopharyngeal tissues, promoting oxidative stress, inflammatory responses, and DNA damage. Chronic exposure overwhelms cellular antioxidant defense systems and promotes malignant transformation.

Environmental exposures can induce epigenetic modifications that alter tumor suppressor gene expression and DNA repair capacity. These changes may be heritable and contribute to familial clustering of NPC cases in high-risk populations.

Understanding the multi-factorial pathogenesis enables the development of personalized risk assessment tools that integrate genetic testing, environmental exposure assessment, and viral status evaluation to identify high-risk individuals for enhanced surveillance and prevention interventions (**Supplementary Figure 1**). Reduction of preserved food consumption and increased fresh fruit and vegetable intake can significantly reduce nitrosamine exposure and provide protective antioxidants. Improved ventilation systems and clean cooking technologies can reduce indoor air pollution exposure in traditional communities. Identification of high-risk genetic profiles enables targeted lifestyle interventions and enhanced screening protocols for affected families.

## 4 Future directions

NPC in Northeast India arises from a complex interplay of genetic predisposition, EBV infection, and distinctive environmental exposures. Recognizing these regional risk factors provides a strong foundation for targeted prevention, early detection, and personalized treatment strategies.

Future efforts should focus on molecular characterization and biomarker development to refine risk stratification and guide therapy of NPC. NPC being a very region-specific cancer, requires region-specific screening that integrates EBV DNA quantification and host genetic markers such as HLA-B*46:01 and rs2860580. In parallel, HPV has been consistently reported in association with NPC, particularly Type I NPC and predominantly in Western populations; however, these observations are largely epidemiological and statistical in nature. Robust mechanistic studies are required to delineate whether HPV plays a causal or co-factorial role in NPC pathogenesis, to define its interaction with EBV where relevant, and to elucidate the molecular pathways through which HPV may contribute to epithelial transformation. Besides, in the Indian context, HPV-positive OPSCC are increasingly recognized in urban centres, with reported HPV positivity rates of 20-40%, substantially lower than the 60-80% reported in Western settings, a disparity likely reflecting differences in HPV transmission patterns and sexual behaviour (8). Genetic susceptibility to HPV-driven HNC, including HLA-DRB1 variants, remains poorly characterized in Indian populations and require dedicated epidemiological studies.

In regard to EBV, In India and other low- and middle-income settings, primary EBV infection typically occurs in early childhood, often within the first few years of life, likely reflecting higher household density, close interpersonal contact, and earlier oral exposure. Serological studies from Indian cohorts demonstrate that EBV infection is acquired early, with high seroprevalence already detectable in young children and near-universal seropositivity by late childhood or adolescence (50, 52). Recent Indian pediatric serology data also show high early exposure, with EBV seroprevalence of ~75–80% among children in a hospital-based cohort, confirming that most infections occur before adolescence (52). In mixed-age Indian populations, overall EBV IgG seropositivity has been reported to be ~89%, with antibodies detectable across nearly all age groups, indicating near-universal exposure by late childhood or early adulthood (53) (53). Consequently, primary infection in adolescents, the setting in which classical infectious mononucleosis most commonly occurs, is relatively uncommon in the Indian population, and clinical infectious mononucleosis is rarely diagnosed. This early-acquisition pattern has important implications for prophylactic EBV vaccination strategies, as the window for immunization prior to natural infection may be considerably narrower in India than in Western countries. mRNA-based EBV vaccines targeting gp350 and gH/gL are currently in Phase I/II trials (54). While universal vaccination may not be feasible given near-ubiquitous childhood infection, targeted immunization of high-risk individuals identified by HLA haplotyping (A2-B46 carriers in Northeastern India) as well as polymorphisms in detoxification and DNA-repair genes such as GSTM1, GSTT1, and XRCC1, particularly in Asian population remains a conceptually compelling preventive strategy that requires evaluation.

Community-based interventions addressing modifiable lifestyle factors, reducing betel quid use, improving indoor air quality through cleaner cooking fuels, and discouraging smoked meat consumption, should be implemented as policies. Although no EBV vaccine currently exists, immunization approaches targeting viral proteins such as LMP1 remain a promising avenue. Integrating molecular surveillance with culturally sensitive public health outreach and longitudinal cohort studies will be essential to reduce NPC incidence and improve outcomes across high-risk Northeastern Indian populations.
