# Supplementary material for: The triad of nasopharyngeal carcinoma pathogenesis: host genetics, viral infection, and environmental exposures
Source: Front Oncol. 2026 Apr 15;16:1800709. doi: 10.3389/fonc.2026.1800709 (PMC13124491; doi:10.3389/fonc.2026.1800709)
Supplement: Supplementary file 1 [file DataSheet1.docx]

Supplementary Material

**SUPPLEMENTARY FIGURES**


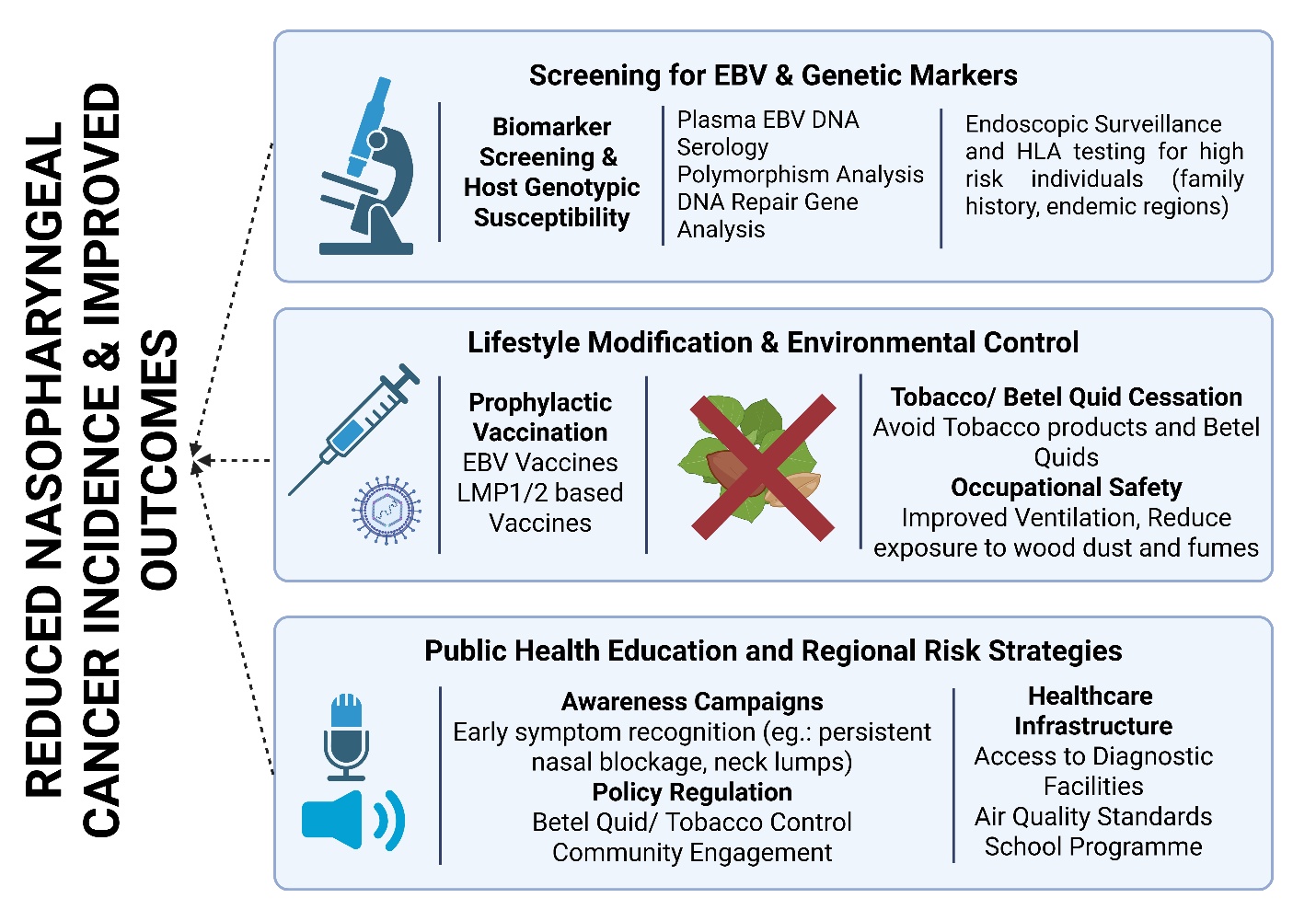


**Figure S1**: Visual guide to prophylactic and risk mitigation strategies against nasopharyngeal carcinoma in India. It outlines a four-step pathway: (a) Screening for EBV and genetic markers (b) Vaccination (future potential) (c) Lifestyle changes like reducing betel quid use and indoor pollution (d) Public health education and regional risk reduction

##
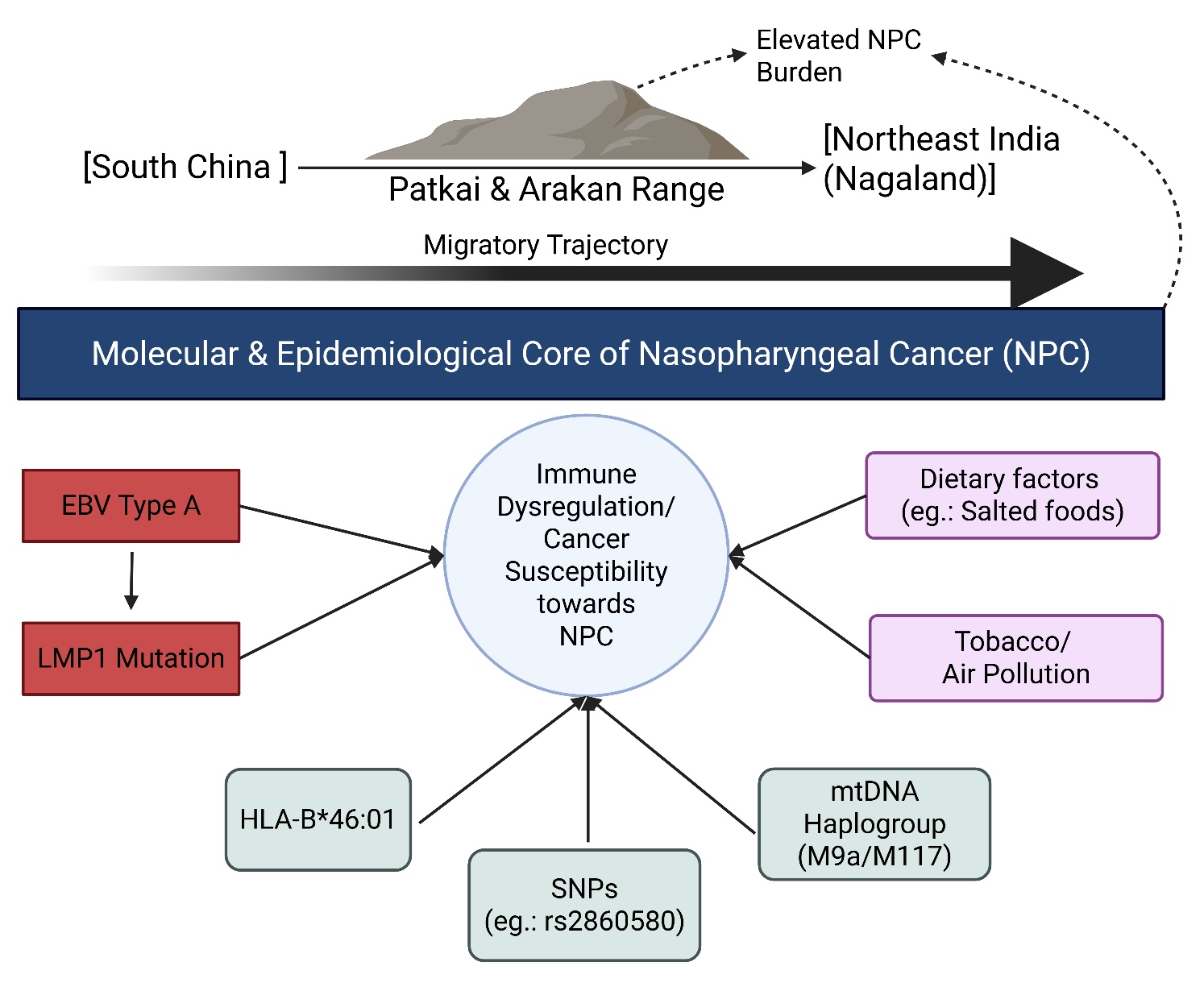


**Figure S2:** Integrative Map Linking Migration and Nasopharyngeal Carcinoma Susceptibility in Northeast India

Systems biology map depicts the hypothesized migratory trajectory from South China (Yunnan/Guangxi) to Northeast India (Nagaland), overlaid with molecular, epidemiological, and anthropological data relevant to NPC. Mitochondrial DNA and Y-chromosome analyses have demonstrated close genetic affinity between Tibeto-Burman–speaking populations of Northeast India and populations of southern China and Southeast Asia, with high frequencies of East-Asian-associated haplogroups, including Y-haplogroup O lineages, supporting models of migration across the eastern Himalayan corridor (1). Host genetic susceptibility is represented by the distribution of HLA class I alleles strongly associated with NPC in endemic southern Chinese populations, particularly HLA-A02:07 and HLA-B46:01, which have also been reported in Tibeto-Burman–derived populations and are thought to contribute to differential immune control of EBV infection (2,3). A lineage and the presence of NPC-associated LMP1 deletion polymorphisms, which are highly prevalent in southern Chinese endemic regions and have also been reported in NPC cases from Northeast India, suggesting circulation of related EBV variants within genetically related host populations (4,5). These viral polymorphisms are believed to influence oncogenic potential through altered NF-κB activation, immune evasion, and latency regulation. Environmental and cultural factors are also represented, including traditional dietary practices involving preserved or smoked foods with high nitrosamine content, which have been implicated in NPC pathogenesis in southern China and in tribal populations of Northeast India.

## SUPPLEMENTARY TABLES

## Table S1: Epidemiology of NPC in North-East India

| **Region/District** | **AAR (per 100,000)** | **Remarks** | **Reference** |
| --- | --- | --- | --- |
| Kohima, Nagaland | 19.4 | Highest recorded in India | Kataki et al. (2011) (6) |
| Imphal West, Manipur | 7.4 | NPC is 4th most common cancer | Kataki et al. (2011) (6) |
| Nagaland | 4.3 | Highest incidence among NE states | Chelleng et al. (2000) (7) |
| Manipur | 5.4 | High prevalence | Kataki et al. (2011) (6) |
| Mizoram | 3.5 | NPC among top head and neck cancers | Kataki et al. (2011) (6) |
| Sikkim | 4.06 | NPC reported as common | Kataki et al. (2011) (6) |
| Assam | 0.6 | Lowest incidence in NE | Kataki et al. (2011) (6) |
| NE Region Combined | ~5–20 | Variable based on district | Kataki et al. (2011) (6); Ghosh SK et al. (2014) (8) |

### ****Table S2: Genetic Susceptibility and HLA Associations in NPC****

| **Genetic Marker** | **Association** | **Population Studied** | **Reference** |
| --- | --- | --- | --- |
| STEAP3 rs138941861 | Risk variant (impacts p53 pathway) | Nagaland, Mizoram, Manipur | Laskar S et al. (2022) (9) |
| JAG1 rs2273059 | Risk variant | Nagaland, Mizoram, Manipur | Laskar S et al. (2022) (9) |
| PARP4 rs17080653 | Protective variant | Nagaland, Mizoram, Manipur | Laskar S et al. (2022) (9) |
| TGFBR1 rs11568778 | Protective variant | Nagaland, Mizoram, Manipur | Laskar S et al. (2022) (9) |
| TP53 rs1042522 | Risk allele | NPC patients worldwide | Laskar S et al. (2020) (10) |
| MDM2 rs2279744 | Risk allele | NPC patients worldwide | Laskar S et al. (2020) (10) |
| CDKN1A rs1059234 | Risk allele | NPC patients worldwide | Laskar S et al. (2020) (10) |
| CYP2A6 polymorphism | Reduces NPC risk (deficient activity) | Manipur | Lourembam DS et al. (2015) (11) |
| CYP1A1 + GSTM1 Null | High risk in smokers (OR=6.42) | Northeast India | Choudhury JH et al. (2015) (12) |
| CDKN2A deletion | Present in ~40% of tumors | NPC patients worldwide | Paul P et al. (2018) (13) |
| p53 codon 72 (Arg > Pro) | Increased NPC risk | Northeast India | Sahu SK et al. (2016) (14) |
| GSTM1/GSTT1 null | Higher risk in smokers/chewers | Nagaland, Mizoram, Manipur | Singh SA et al. (2019) (15) |
| XRCC1 Arg399Gln | 2.76–3.2× increased risk | Northeast India | Singh SA et al. (2016) (16) |
| BAX -248G>A | Higher risk (reduced apoptosis) | Northeast India | Chatterjee K et al. (2021) (17) |
| HLA-D6S2704 (allele 218) | Risk variant | Northeast India | Lakhanpal M et al. (2015) (18) |
| HLA-HL003 (allele 121) | Risk variant | Northeast India | Lakhanpal M et al. (2015) (18) |
| HLA-HL003 (allele 127) | Protective variant | Northeast India | Lakhanpal M et al. (2015) (18) |
| TNF-β GG genotype | Protective | Northeast India | Lakhanpal M et al. (2015) (19) |
| TNF-β AG genotype | Increased susceptibility | Northeast India | Lakhanpal M et al. (2015) (19) |
| HSP70 +2437 CC genotype | Protective | Northeast India | Lakhanpal M et al. (2015) (19) |

## Table S3: Epstein-Barr Virus and Molecular Pathogenesis

| **Marker/Pathway** | **Findings** | **Population** | **Reference** |
| --- | --- | --- | --- |
| EBNA1 | Positive in 92.5% of NPC samples | Nagaland | Borthakur P et al. (2016) (20) |
| EBNA1 | Positive in 40.8 % of NPC samples | NE India | Chatterjee K et al.(2022) (21) |
| EBNA2 | Positive in 0.8 % of NPC samples | NE India | Chatterjee K et al.(2022) (21) |
| LMP1 | Positive in 72.8% of NPC samples | Manipur, Nagaland, Mizoram | Singh SA et al. (2014) (22) |
| LMP1 | Positive in 90% of NPC samples | Nagaland | Borthakur P et al. (2016) (20) |
| High EBV DNA Load | Associated with advanced NPC stage | NE India | Lourembam DS et al.(2015) (11) |
| PI3K, p53 Pathways | Variants observed in patients | NE India | Laskar S et al.(2020) (10) |
| EBER (CISH) | 59% positivity; assoc. with smoked food and Naga ethnicity | NE India | Saikia A et al. (2016) (23) |
| EBV (*LMP1*  gene) | EBV infection linked to GST null genotype & mtDNA alterations also 2.17 fold increased risk of NPC | Manipur,Nagaland,Mizoram | Ghosh SK et al. (2014) (8) |
| EBNA1 | Positive in 87.86% of NPC samples | Manipur,Nagaland | Roy Chattopadhyay N et al. (2019) (24) |
| EBNA2 | Positive in 9.94% of NPC samples | Manipur,Nagaland | Roy Chattopadhyay N et al.(2019) (24) |

### ****Table S4: Environmental and Lifestyle Risk Factors for NPC****

| **Category** | **Risk Factor** | **Association with NPC** | **Key References** |
| --- | --- | --- | --- |
| **Dietary** | Smoked/salted/fermented foods | OR = 2.0–10.8 (highest for smoked meat) | Ghosh SK et al. (2014) (8) |
|  | Fermented fish intake | OR = 2.07–4.34 | Singh SA et al. (2016) (16) |
|  | Preserved foods (NDMA/BaP*) | Mutagenic; linked to NPC in Nagaland | Roy Chattopadhyay et al. (2017) (25) |
| **Tobacco/Alcohol** | Smoking | OR = 4.5–37.49 (highest in smokers) | Laskar S et al. (2022) (9) |
|  | Betel quid chewing | OR = 2.77–7.0 | Singh SA et al. (2016) (16) |
|  | Alcohol consumption | OR = 2.11–2.13 | Lakhanpal M et al. (2015) (27) |
| **Household** | Firewood cooking | OR = 3.40–12.07 | Keppen C et al. (2020) (26) |
|  | Poor ventilation/mud houses | OR = 3.46–3.63 | Lakhanpal M et al. (2015) (18) |
|  | Kerosene/mosquito repellent | Lower 5-year survival in NPC patients | Chatterjee K et al. (2022) (21) |
| **Gene-Environment** | XRCC1 + smoked meat | OR = 4.07–7.47 | Singh SA et al. (2016) (16) |
|  | CYP1A1 CC + smoking | OR = 7.55–10.8 (head/neck cancer) | Singh SA et al. (2015) (27) |
| **Other** | Herbal medicine/nasal use | OR = 3.18–21.9 | Chelleng PK et al. (2000) (7) |

NDMA: N-nitrosodimethylamine; BaP: Benzo(a)pyrene

**Table S5: NPC Histological subtypes in North East India**

| **Population** | **Predominant Histological Subtype** | **% WHO type** | **Reference** |
| --- | --- | --- | --- |
| Manipur | Type III | 75 | Sharma TD et al.(2011) (28) |
| Manipur | Type III | 83 | Lourembam DS et al.(2015) (11) |
| NE India | Type III | 57.6 | Chatterjee K et al.(2022) (21) |
| Nagaland | Type III | 45 | Borthakur P et al. (2016) (20) |
| NE India | Type III | 65 | Lakhanpal M et al.(2015) (18) |
| NE India | Type III | 88 | Saikia A et al. (2016) (23) |
| NE India | Type I | 55.7 | Sahu SK et al. (2016) (14) |

## Table S6: Gender & Age Distribution by Study

| **Study** | **Gender Distribution** | **Age Distribution** |
| --- | --- | --- |
| Singh SA et al. (2019) (24) | M:F = 1.46:1 | ≤ 50 yrs=65.1%  > 50 yrs=34.9% |
| Singh SA et al.(2015) (27) | M:F = 2.1:1 | ≤ 50 yrs= 44.7% |
| Sharma TD et al.(2011) (28) | M:F = 2.2:1 | Mean Age: (49.7 ± 10.7 yrs). |
| Lourembam DS et al.(2015) (11) | M:F = 2.2:1 | Mean Age: 48.9 (range, 18-80) years |
| Borthakur P et al. (2016) (20) | M:F= 3.44:1 | Mean Age :  (50±12.78 yrs) |
| Singh SA et al. (2014) (22) | M:F= 3.38:1 | ≤ 50 yrs=37.1%  > 50 yrs=62.9% |
| Lakhanpal M et al.(2015) (18) | M:F= 3.8:1 | Mean Age: 48 (range, 12-80) years |
| Ghosh SK et al. (2014) (8) | M:F= 3.26:1 | ≤ 50 yrs=35.9%  > 50 yrs=64% |
| Saikia A et al. (2016) (23) | M:F= 2.4:1 | Mean Age: 44.64 years |
| Sahu SK et al. (2016) (14) | M:F=1.91:1 | ≤ 50 yrs=64.2%  > 50 yrs=35.7% |
| Keppen C et al. (2020) (26) | M:F=2.36:1 | ≤ 50 yrs=70.3%  > 50 yrs=29.7% |
| Lakhanpal M et al. (2016) (19) | M:F=3.8:1 | ≤ 50 yrs=20%  > 50 yrs=80% |
| Laskar S et al. (2022) (9) | M:F = 1.9:1 | Mean Age: (46.58 ± 1.39 yrs) |

**REFERENCES**

1. Borkar M, Ahmad F, Khan F, Agrawal S. Paleolithic spread of Y-chromosomal lineage of tribes in eastern and northeastern India. *Annals of Human Biology* (2011) 38:736–746.

2. Cordaux R, Weiss G, Saha N, Stoneking M. The Northeast Indian passageway: A barrier or corridor for human migrations? *Mol Biol Evol* (2004) 21:1525–1533. doi: 10.1093/molbev/msh151

3. Tagore D, Majumder PP, Chatterjee A, Basu A. Multiple migrations from East Asia led to linguistic transformation in NorthEast India and mainland Southeast Asia. *Front Genet* (2022) 13:1–11. doi: 10.3389/fgene.2022.1023870

4. Singh Lourembam D, Ramsing Singh T, Shanjukumar Singh L, Author C. IL-16 and XRCC1 gene polymorphism along with hypoalbuminemia correlates with the risk and prognosis of NPC among the ethnic population of northeast region of India. *IOSR Journal of Biotechnology and Biochemistry (IOSR-JBB* 3:45–53. doi: 10.9790/264X-03054553

5. Zhang X, Song K, Mai H, Jia W, Feng B, Xia J, et al. The 30-bp deletion variant: a polymorphism of latent membrane protein 1 prevalent in endemic and non-endemic areas of nasopharyngeal carcinomas in China. *Cancer Lett* (2002) 176:65–73.

6. Kataki AC, Simons MJ, Das AK, Sharma K, Kumar Mehra N. Nasopharyngeal carcinoma in the Northeastern states of India. *Chin J Cancer* (2011) 30:106–113. doi: 10.5732/cjc.010.10607

7. Chelleng P, Narain K, Das H, Chetia M, Mahanta J. Risk factors for cancer nasopharynx: a case-control study from Nagaland, India. *National Medical Journal of India* (2000) 13:6–8.

8. Ghosh S, Singh A, Mondal R, Kapfo W, Khamo V, Singh Y. Dysfunction of mitochondria due to environmental carcinogens in nasopharyngeal carcinoma in the ethnic group of Northeast Indian population. *Tumor Biology* (2014) 35:6715–24. doi: 10.1007/s13277-014-1897-x

9. Laskar S, Das R, Kundu S, Saha A, Nandi N, Choudhury Y, Kumar Ghosh S. Whole exome sequencing identifies the potential role of genes involved in p53 pathway in Nasopharyngeal Carcinoma from Northeast India. *Gene* (2022) 812:146099. doi: 10.1016/j.gene.2021.146099

10. Laskar S, Kundu S, Das R, Choudhury Y, Ghosh SK. Clinically significant variants associated with nasopharyngeal carcinoma: Findings of a meta-analysis study. *Meta Gene* (2020) 24:100688. doi: 10.1016/j.mgene.2020.100688

11. Lourembam DS, Singh AR, Sharma TD, Singh TS, Singh TR, Singh LS. Evaluation of risk factors for nasopharyngeal carcinoma in a high-risk area of India, the Northeastern region. *Asian Pacific Journal of Cancer Prevention* (2015) 16:4927–4935. doi: 10.7314/APJCP.2015.16.12.4927

12. Choudhury J, Singh S, Kundu S, Choudhury B, Talukdar F, Srivasta S, Laskar R, Dhar B, Das R, Laskar S, et al. Tobacco carcinogen-metabolizing genes CYP1A1, GSTM1, and GSTT1 polymorphisms and their interaction with tobacco exposure influence the risk of head and neck cancer in Northeast Indian population. *Tumor Biology* (2015) 36:5773–83. doi: 10.1007/s13277-015-3246-0

13. Paul P, Deka H, Malakar AK, Halder B, Chakraborty S. Nasopharyngeal carcinoma: Understanding its molecular biology at a fine scale. *European Journal of Cancer Prevention* (2018) 27:33–41. doi: 10.1097/CEJ.0000000000000314

14. Sahu SK, Chakrabarti S, Roy SD, Baishya N, Reddy RR, Suklabaidya S, Kumar A, Mohanty S, Maji S, Suryanwanshi A, et al. Association of p53 codon72 Arg>Pro polymorphism with susceptibility to nasopharyngeal carcinoma: Evidence from a case-control study and meta-analysis. *Oncogenesis* (2016) 5: doi: 10.1038/oncsis.2016.31

15. Singh SA, Ghosh SK. Metabolic Phase I (CYPs) and Phase II (GSTs) Gene Polymorphisms and Their Interaction with Environmental Factors in Nasopharyngeal Cancer from the Ethnic Population of Northeast India. *Pathology and Oncology Research* (2019) 25:33–44. doi: 10.1007/s12253-017-0309-0

16. Singh SA, Ghosh SK. Polymorphisms of XRCC1 and XRCC2 DNA repair genes and interaction with environmental factors influence the risk of nasopharyngeal carcinoma in northeast India. *Asian Pacific Journal of Cancer Prevention* (2016) 17:2811–2819.

17. Chatterjee K, De S, Roy SD, Sahu SK, Chakraborty A, Ghatak S, Das N, Mal S, Chattopadhyay NR, Das P, et al. BAX -248 G>A and BCL2 -938 C>A Variant Lowers the Survival in Patients with Nasopharyngeal Carcinoma and Could be Associated with Tissue-Specific Malignancies: A Multi-Method Approach. *Asian Pacific Journal of Cancer Prevention* (2021) 22:1171–1181. doi: 10.31557/APJCP.2021.22.4.1171

18. Lakhanpal M, Singh L, Rahman T, Sharma J, Singh M, Kataki A, Verma S, Chauhan P, Singh Y, Wajid S, et al. Contribution of susceptibility locus at HLA class I region and environmental factors to occurrence of nasopharyngeal cancer in Northeast India. *Tumor Biology* (2015) 36:3061–73.

19. Lakhanpal M, Singh LC, Rahman T, Sharma J, Singh MM, Kataki AC, Verma S, Pandrangi SL, Singh YM, Wajid S, et al. Study of single nucleotide polymorphisms of tumour necrosis factors and HSP genes in nasopharyngeal carcinoma in North East India. *Tumor Biology* (2016) 37:271–281. doi: 10.1007/s13277-015-3767-6

20. Borthakur P, Kataki K, Keppen C, Khamo V, Deka M. Expression of Epstein Barr Virus Encoded EBNA1 and LMP1 Oncoproteins in Nasopharyngeal Carcinomas from Northeast India. *Asian Pacific Journal of Cancer Prevention* (2016) 17:3411–3416.

21. Chatterjee K, Roy SD, Chakraborty K, Haque A, Chakrabarti S, Mukherjee S, Mal S, Das N, Sahu SK, Chattopadhyay NR, et al. Lifestyle, Epstein-Barr virus infection, and other factors could impede nasopharyngeal cancer survivorship: a five-year cross-sectional study in North Eastern India. *Virusdisease* (2022) 33:371–382. doi: 10.1007/s13337-022-00789-5

22. Anil Singh S, Ghosh S. Association of Epstein Barr virus and lifestyle on nasopharyngeal cancer risk among the ethnic population of northeast India. *Sci Technol J* (2014) 2:95–102.

23. Saikia A, Raphael V, Shunyu NB, Khonglah Y, Mishra J, Jitani AK, Medhi J. Analysis of Epstein Barr Virus Encoded RNA Expression in Nasopharyngeal Carcinoma in North-Eastern India: A Chromogenic in Situ Hybridization Based Study. *Iran J Otorhinolaryngol* (2016) 28:267–274. doi: 10.22038/IJORL.2016.7036

24. Roy Chattopadhyay N, Chakrabarti S, Chatterjee K, Deb Roy S, Kumar Sahu S, Reddy RR, Das P, Bijay Kanrar B, Kumar Das A, Tsering S, et al. Histocompatibility locus antigens regions contribute to the ethnicity bias of Epstein-Barr virus-associated nasopharyngeal carcinoma in higher-incidence populations. *Scand J Immunol* (2019) 90:1–13. doi: 10.1111/sji.12796

25. Roy Chattopadhyay N, Das P, Chatterjee K, Choudhuri T. Higher incidence of nasopharyngeal carcinoma in some regions in the world confers for interplay between genetic factors and external stimuli. *Drug Discov Ther* (2017) 11:170–180. doi: 10.5582/ddt.2017.01030

26. Keppen C, Barooah P, Borthakur P, Saikia S, Deka M, Bhattacharjee S, Keppen J, Khamo V, Medhi S. Genetic Polymorphisms Along with Dietary and Environmental Factors Enhance the Susceptibility to Nasopharyngeal Carcinoma in Nagaland of Northeast India. *Biochem Genet* (2020) 58:533–550. doi: 10.1007/s10528-020-09954-1

27. Singh SA, Choudhury JH, Kapfo W, Kundu S, Dhar B, Laskar S, Das R, Kumar M, Ghosh SK. Influence of the CYP1A1 T3801C polymorphism on tobacco and alcohol-associated head and neck cancer susceptibility in Northeast India. *Asian Pacific Journal of Cancer Prevention* (2015) 16:6953–6961. doi: 10.7314/APJCP.2015.16.16.6953

28. Dhaneshor Sharma T, Tomcha Singh T, Laishram RS, Durlav Chandra Sharma L, Sunita AK, Tiameren Imchen L. Nasopharyngeal carcinoma - A clinico-pathological study in a regional cancer centre of northeastern India. *Asian Pacific Journal of Cancer Prevention* (2011) 12:1583–1587.
